# Endovascular Repair of Juxtarenal and Pararenal Abdominal Aortic Aneurysms Using a Novel Low-Profile Fenestrated Custom-Made Endograft: Technical Details and Short-Term Outcomes

**DOI:** 10.1177/15266028241227392

**Published:** 2024-01-30

**Authors:** K. K. Yeung, J. H. Nederhoed, B. L. Tran, S. Di Gregorio, G. Pratesi, M. Bastianon, C. Melani, V. Riambau, T. Bloemert-Tuin, C. E. V. B. Hazenberg, J. A. van Herwaarden, R. Balm, R. J. Lely, B. B. van der Meijs, J. D. Blankensteijn, A. W. J. Hoksbergen, V. Jongkind

**Affiliations:** 1 Department of Surgery, Amsterdam UMC, University of Amsterdam, Amsterdam, The Netherlands; 2 Department of Surgery, Amsterdam UMC, Vrije Universiteit Amsterdam, Amsterdam, The Netherlands; 3 Amsterdam Cardiovascular Sciences, Microcirculation, Atherosclerosis & Ischemic Syndromes, Amsterdam, The Netherlands; 4 Department of Surgical Sciences and Integrated Diagnostics, University of Genoa, Genoa, Italy; 5 IRCCS Ospedale Policlinico San Martino, Genoa, Italy; 6 Angiology and Vascular Surgery, Cardiovascular Institute, Hospital Clinic of Barcelona, Barcelona, Spain; 7 Department of Vascular Surgery, University Medical Center Utrecht, Utrecht, The Netherlands; 8 Department of Interventional Radiology, Amsterdam UMC, Vrije Universiteit Amsterdam, Amsterdam, The Netherlands

**Keywords:** fenestrated TREO, endograft, abdominal aortic aneurysm, endovascular, TERUMO aortic

## Abstract

**Introduction:**

The aim of this study is to share preliminary experiences and outcomes with a novel custom-made fenestrated TREO^®^ Abdominal Stent-Graft System to treat juxtarenal and pararenal abdominal aortic aneurysms (AAAs).

**Methods:**

Juxtarenal and pararenal AAA patients treated with the custom-made fenestrated TREO^®^ Abdominal Stent-Graft System were included from 4 high-volume European academic medical centers from June 2021 to September 2023. Technical success and 30-day/in-hospital mortality and complications were analyzed. Technical success was defined as successful endovascular implantation of the stent graft with preservation of antegrade flow to the target vessels, and absence of type 1 or 2 endoleak (EL) at the first postoperative computed tomography angiography (CTA).

**Results:**

Forty-two consecutive patients were included. The majority of the devices were constructed with 2 (N=4; 9.5%), 3 (N=9; 21.4%), or 4 (N=27; 64%) fenestrations. In 1 case, the device was constructed with a single fenestration (2.4%) and 1 device contained 5 fenestrations (2.4%); 17% had previous AAA repair. Target vessel cannulation with placement of a bridging stent was successful in all but 1 vessel (99, 3%). One aneurysm-related death occurred in the direct postoperative period and 2 limb occlusions necessitated reintervention during admission. In the median follow-up period of 101 (2–620) days, 3 more patients died due to non–aneurysm-related causes. Technical success was achieved in 90% of the cases. Nineteen ELs were seen on the first postoperative CT scan: 1 type 1b EL (N=1; 2%), 15 type 2 ELs (N=15; 36%), and 3 type 3 ELs (N=3%). Eleven patients received more than 1 CT scan during a median follow-up of 361 days (82-620): 3 type 2 ELs resolved and 1 type 3 EL was treated in this period. In the follow-up, 1 patient had a coagulation disorder that caused occlusions of the branches.

**Conclusion:**

The results of the first experiences using the custom-made fenestrated TREO® Abdominal Stent-Graft System in Europe are promising. There was a low short-term mortality and morbidity rate in these patients of which 17% had previous AAA repair. Mid-term and long-term follow-up data are needed to evaluate endograft durability and performance.

**Clinical Impact:**

This study shows the first experiences and short-term results of a novel low-profile custom-made device: the custom-made fenestrated TREO^®^ Abdominal Stent-Graft System. Showing these results and experiences can help the physicians in clinical decision-making for their patients.

## Introduction

In the last 2 decades, endovascular repair of aortic aneurysms^1^ has rapidly advanced and evolution in design has led to fenestrated endovascular repair for more complex aortic aneurysms, such as juxtarenal aortic aneurysm (JAA) and pararenal aortic aneurysm (PAA).^2^ One of the relatively new devices is the low-profile custom-made fenestrated TREO^®^ endograft (19Fr OD; TERUMO Aortic, Bolton Medical Inc., USA). This device could offer easier iliac access and navigation, which in turn could result in a more controlled deployment for accurate placement. The design includes an option for an unsupported part at the visceral and renal level in the main body of the endograft, enhancing flexibility at the fenestrations. It could diminish limitations in positioning of fenestrations, especially if the patients received a previous aortic repair.

This study aims to describe the details and usage of the lower profile fenestrated TREO^®^ endograft and share preliminary outcomes from multiple academic medical centers in Europe.

## Materials and Methods

The fenestrated TREO^®^ endograft was first implanted in European sites that had experience with the TREO^®^ endograft or were running the TIGER registry, a prospective registry for aortic endografts from TERUMO Aortic (ClinicalTrials.gov Identifier: NCT04246463; Bolton Medical Inc., USA). The first experiences and short-term outcomes of 4 European academic medical centers using this fenestrated TREO^®^ endograft were analyzed. The latter centers are treating at least 50 complex aortic surgeries a year. Next to the fenestrated TREO^®^ endograft, other brands like custom-made devices from Cook were used as well. We included in this study only consecutive patients receiving the fenestrated TREO^®^ endograft from June 2021 to September 2023 (Amsterdam: 17/42; Italy: 9/42; Utrecht: 8/42; and Barcelona: 8/42). The study was conducted following the local guidelines of the ethical commission of the specific site. Technical success was defined as successful endovascular implantation of the stent graft with preservation of antegrade flow to the target vessels, and absence of type 1 or 2 endoleak (EL) at the first postoperative computed tomography angiography (CTA).

### Operating Procedure

**Figure 1.**
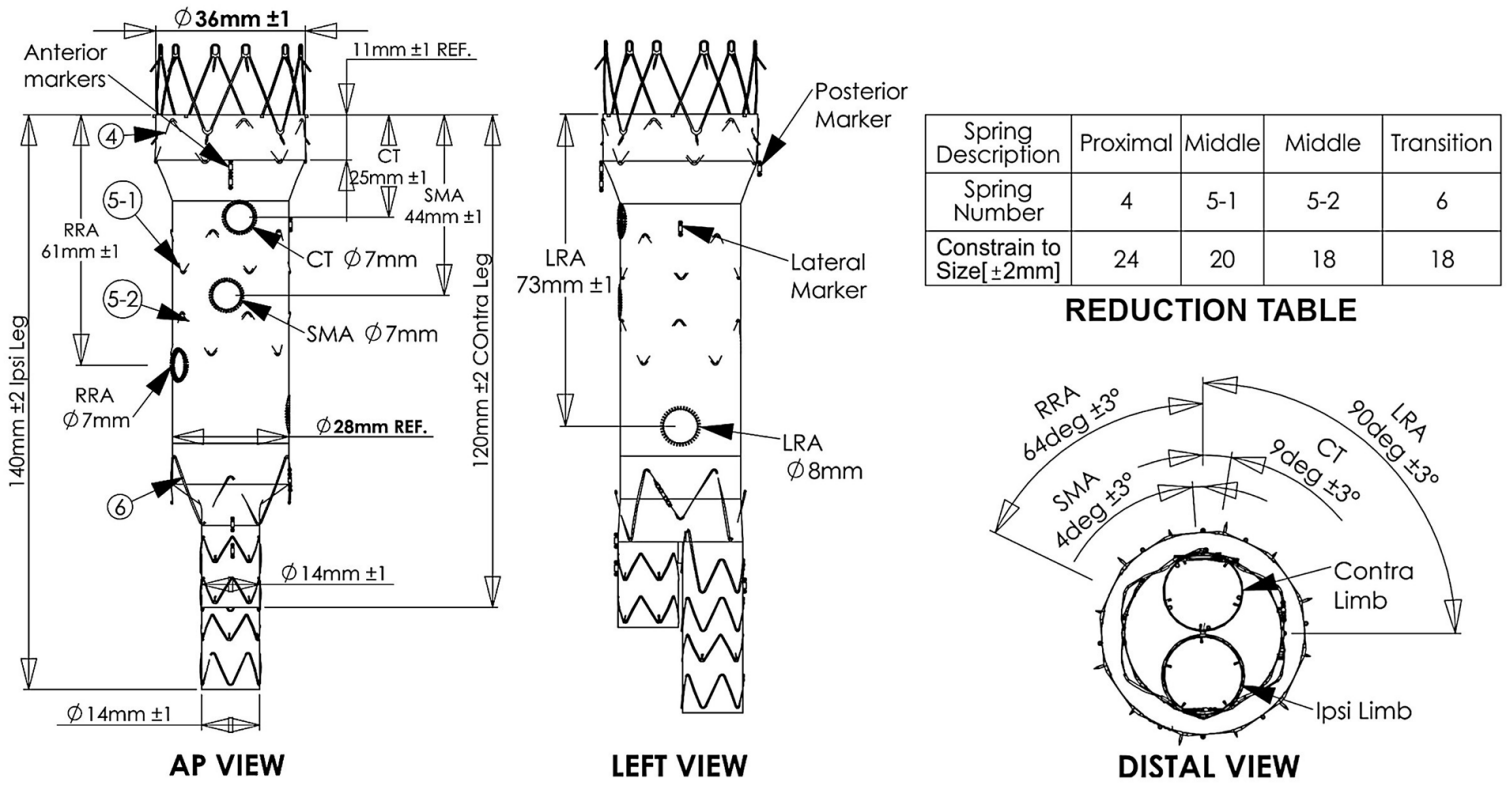
Custom sketch (graft-plan) adjusted on the patient’s anatomy. The arrows indicate the markers and the fenestrations. mm, millimeters; RRA, right renal artery; CT, celiac trunk; SMA, superior mesenteric artery; AP, anterior-posterior; LRA, left renal artery; deg, degrees, REF, reference.

**Figure 2.**
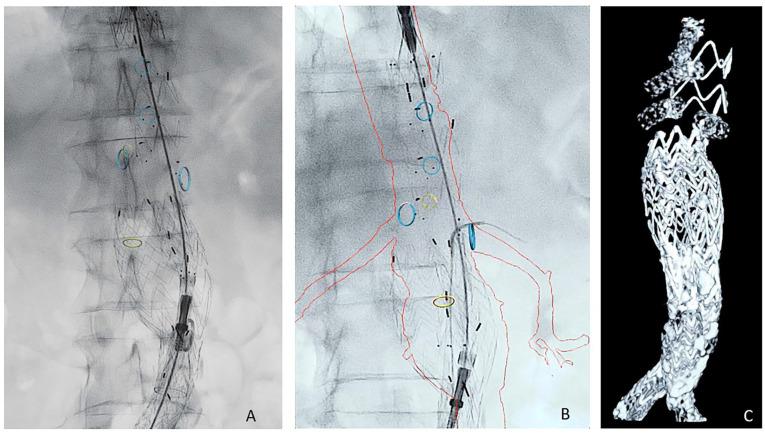
Treatment of a type 1a endoleak by placement of fenestrated TREO endograft in an earlier infrarenal abdominal endograft. (A) Partial deployment of the fenestrated TREO endograft, while focusing on the fenestrations (using the overlay of the vessel navigator), and anterior and posterior markers on the fenestrated TREO endograft. (B) Cannulation of the left renal artery. (C) SMART-CT at the end of the procedure showing the final result. CT, computed tomography.

A custom-made sketch (graft plan) of the custom-made fenestrated TREO^®^ endograft is made based on the anatomy of the patient (Figure 1). On the day of the surgery, following access preparation, the 19 French custom-made main device (bifurcation device; fenestrated TREO^®^ endograft) is introduced from the right or left common femoral artery. Diagnostic angiography is performed to mark the origin of the renal arteries and the device is deployed up to the contralateral leg. based on the position of (one of) these arteries, anteroposterior markers and the marker at the left side of the endograft as well as the fenestration of the renal artery guide a proper positioning of the graft (Figures 1 and 2A). A vessel navigator (Philips Medical, the Netherlands) can be used as an overlay indicating the target vessels (Figure 2A). After first deployment, the endograft is still constrained to 20 mm by circular diameter reducing ties, making it easy to maneuver the endograft, and adjust the position of the fenestration before the target branches (Figure 2A). After this partial deployment and cannulation of the contralateral limb of the endograft, a 14-20 Fr sheath is placed in the contralateral limb. Thereafter, cannulation of the fenestrations using a curved catheter or steerable sheath is performed (Figure 2B). The wire can be left in the target vessel or 7 Fr sheaths of 55 cm are then placed in the target vessels through the fenestration. Now, the diameter-reducing ties can be released and the sealing zones ballooned. Balloon expandable stent grafts are used as bridging stents and consequently flared in the fenestrations (10 or 12×20 mm balloon). After final extension in the iliac limbs, the final angiography or cone-beam computed tomography (CT) can be performed to evaluate the results (Figure 2C).

### Details of the Custom-Made Fenestrated TREO^®^ Endograft

All stent grafts comprised self-expanding nitinol stents sutured to woven polyester fabric. The sutures composition is made of polytetrafluoroethylene (PTFE)-impregnated polyester. The markers applied for fluoroscopic visualization are made of platinum-iridium. The fenestrations are reinforced with closed circumferential nitinol rings sutured around the fenestration hole with the markers direct at the edge of the holes without any spacing in between. The fenestrated TREO^®^ endograft is a custom-made stent graft specifically designed to the anatomy of the patient with up to 5 fenestrations possible. The length of coverage proximal to the celiac is on average 23 to 25 mm above the celiac trunk, which is adjustable to the preference of the surgeon to a minimum of 9 mm. In the design, there will be 3 main regions in the body part that is fully circumferentially reduced by reducing ties:

Proximal sealing part (minimal length: 9 mm. with an average length of ~25 mm. with an oversizing of ~20% limited to a proximal diameter of max. Ø36 mm.
Body part that can be tapered to the anatomy, design to have wall apposition and minimal oversizing at the levels of the fenestrations: up to ~10%. Several sealing areas can be applied in this body part based on the surgeon’s preference.
Transition spring to accommodate the transition of the bifurcation, which is a default of Ø28 mm.

The circumferential reducing ties are design to reduce the whole body part of the graft without changing the designed angles of the fenestrations. Amount of reduction is depended on the anatomy with an average of ~33%. The custom-made fenestrated TREO^®^ endograft is a bifurcated endograft. The bifurcation exists of 2 limbs of each Ø14 mm with “locking stents” to prevent disconnection of the limbs. Maximal total length of the fenestrated TREO is 160 mm.

## Results

A total of 42 patients were analyzed in this study. Thirty-four male and 8 females were treated, with a mean age of 76±6 years. The baseline characteristics of the patients were summarized in Table 1. The mean diameter of the treated aneurysms was 65±11 mm. Four custom-made devices consisted of 2 fenestrations (N=4), 9 of 3 fenestrations (N=9), and the majority of consisted of 4 fenestrations (N=27; 64%), 1 of 5 fenestrations and 1 of 1 fenestration. One patient had an additional fenestrated leg component for the inferior renal polar artery of a horse shoe kidney configuration. In 6 patients (16%), the custom-made fenestrated TREO^®^ endograft was placed in an earlier endovascular aortic repair (EVAR) because of a type 1a EL and in 1 patient in a previous open aortic aneurysm repair with Dacron prosthesis. In total, there were 149 fenestrations distributed among the 4 common target vessels; the celiac trunk (N=30), the superior mesenteric artery (N=38), the right renal artery (N=41), and the left renal artery (N=39) and 1 separate fenestration for the right hypogastric artery.

**Table 1.** Baseline Characteristics: Baseline Patient Characteristics Summarized.

Patient characteristics	Participants (N=42)
Demographics	
	Total N (%)
Age (years)	76±6
Sex	
Male	34 (81)
Female	8 (19)
BMI (kg/m^2^)	27.62±5.03
Comorbidities and risk factors	
Coronary artery disease	18 (43)
Congestive heart failure	4 (710)
Hypertension	31 (74)
Hypercholesterolemia	20 (47)
COPD	12 (29)
Peripheral artery disease	9 (21)
Diabetes mellitus	6 (14)
Stroke or TIA	7 (17)
Chronic kidney disease	5 (12)
Dialysis	0 (0)
Aneurysm history	
Type of aneurysm	
Infrarenal (incl. hostile neck)	6 (14)
Juxtarenal	33 (79)
Pararenal	3 (7)
Previous abdominal aortic repair	7 (17)
Previous abdominal open repair	1 (2)
Previous abdominal endovascular repair	6 (16)
Largest aneurysm diameter (mm)	64.9±11.4
Preoperative laboratory results	
Hemoglobin (mmol/L)	8.7±1.9
eGFR (×10^9/L)	66.3±17.3
Creatinine (μmol/L)	95.7±43.30

Abbreviations: BMI, body mass index; COPD, chronic obstructive pulmonary disease; eGFR, estimated glomerular filtration rate; TIA, transient ischemic attack.

The mean time of the procedure from incision to closure was 244±91 minutes with a mean fluoroscopy time of 98±42 minutes. The target vessels could be cannulated in all but 1 target vessel (N=148/149; 99%) due to the strut of a previous endograft; 77% (N=34/42) of the patients had a percutaneous approach. Seven of 8 cases with open femoral access were performed in 1 center. In 3 patients, there was an additional procedure needed related to bleeding complications of the access: blood leakage because of failure of the puncture closure system needing a cutdown, leakage of the sheath, and surgical exploration of a fem-fem crossover. Median blood loss was 250 mL (50–4500 mL). The completion angiography at the end of the fenestrated endovascular aortic repair (FEVAR) procedure showed a total of 19 ELs (N=19; 45%), evaluated as type 2.

In the postoperative period, 1 patient died within 30 days in the hospital due to an irreversible systemic inflammatory response syndrome (SIRS) reaction after a long operation time (difficulties in cannulation of the superior mesenteric artery due to its steep anatomy) followed by explorative laparotomy (evaluation of bowel ischemia, which was not the case) and anemia that could not be improved with red blood cell transfusions because of his religion. Three other patients required a reintervention: 2 because of limb occlusions and 1 because of a significant stenosis in the limb (n=3; 7%). See Table 2 for a summary of all postoperative complications. The median hospital stay was 3 (1–39) days. All patients received a postoperative CTA scan within 30 days. On the first postoperative CTA scan, there were 19 ELs (N=19; 45%), of which most of them were type 2 ELs (n=15; 35, 7%); there was 1 type 1b EL and 3 type 3 ELs. The median follow-up period consisted of 101 (2–620) days. In this period, 3 other patients died; 2 of them due to cancer (1 progression and 1 newly diagnosed) and 1 unknown cause. Eleven patients received more than 1 CTA during a median follow-up of 361 days (82–620) in the out-patient clinic: 3 type 2 ELs resolved spontaneously without treatment and 1 type 3 EL was treated with ballooning at the fenestration site in this period. Fifty-five percent (6 patients) in the follow-up showed sac shrinkage (>5 mm) and 98% target vessel patency was achieved during follow-up (N=146/149). The occluded target vessels were found in 1 patient with a coagulation disorder, which was not diagnosed or known before the procedure. Patient received successful thrombolysis without signs of a technical failure of the initial FEVAR.

**Table 2.** In-Hospital and 30-day Information: Postoperative Outcomes Information From Patients Treated With the Fenestrated TREO Stent Graft.

Postoperative information	Participants (N=42)
	Total N (%)
In-hospital or within 30 days	
Death	1 (2.4)
Aortic-related death	1 (2.4)
Myocardial infarction	1 (2.4)^a^
Cardiac arrhythmia	1 (2.4)
Congestive heart failure	1 (2.4)
Respiratory failure	2 (4.7)
Pneumonia	4 (9.5)
Stroke	0
Access site complications (i.e., hematoma, false aneurysm)	3 (7.1)
Spinal cord ischemia	1 (2.4)
Acute kidney injury (RIFLE criteria)	1 (2.4)
Dialysis	0
Bowel ischemia	1 (2.4)
Hospital stay (median, min-max)	
From procedure to discharge, in days	3 (1–39)
Reintervention aneurysm-related	4 (9.5)^b^
FIRST postoperative CTA	42 (100)
Total endoleaks	19 (45)
Endoleak type 1b	1 (2.4)
Endoleak type 2	15 (35.7)
Endoleak type 3	3 (7.1)^c^
Limb stenosis	1 (2.4)
Branch stenosis or occlusions	0
Last CTA in follow-up (median 361 days [82-620])	11 (26.2)
Total endoleaks	4 (36)
Endoleak type 1b	0
Endoleak type 2	3 (27)
Endoleak type 3	1 (9)
Sac shrinkage (>5 mm)	6 (55)
Branch stenosis or occlusions	3/149 (2)^d^

Abbreviation: CTA, computed tomography angiography. RIFLE, Risk, Injury, Failure, Loss of Kidney Function, Endstage kidney disease

a Myocardial infarction needing percutaneous cardiac intervention.

b Two reinterventions for femoral artery thrombosis (embolectomy), 1 for stenosis in the limb (relining) and 1 relaparotomy because of SIRS and bowel ischemia.

c One type 3a and 2 type 3c.

d One patient had 3 occluded vessels because of a coagulation disorder.

## Discussion

Our multicenter international study reported the first data and experience with the custom-made fenestrated TREO^®^ endograft in Europe, including 42 patients. Overall, the device was found easy in use and safe. In this study, there was a high success rate for the cannulation of the target vessels and placement of the bridging stents through the fenestrations (99%). The surgeons found that the advantages of the device were the lower profile (19 Fr), the circumferential reducing ties and the choice between a supported or unsupported body at the site of the fenestrations, which might improve the maneuverability of the endograft in kinked anatomy or small access vessels. The main reasons reported by the treating centers (beside surgeon’s preference) was to use the device in more challenging cases i.e. small access, kinked anatomy in the iliac arteries or aortic neck or previous aortic repair, reflected in the fact that 17% of our cohort had a previous abdominal aortic aneurysm (AAA) repair. Therefore, the main complications were also related to access problems or previous vascular surgery (difficulty in cannulation of the renal artery because of the previous bare stent). Iliac tortuosity has been associated with increased rate of reinterventions and increased fluoroscopy time.^3^ Although we did not perform a comparative analysis and we can only present short-term outcomes, in this preliminary study, the technical success rate was high, but indeed high fluoroscopy times were noticed. Low morbidity and mortality rates could be achieved with the fenestrated TREO^®^ endograft. The early results of the custom-made fenestrated TREO^®^ endograft also showed comparable results with previous reports on the outcome of FEVAR.^2,4^ As the short-term outcomes are promising in this study, a high number of type 2 endoleaks persist within the follow-up 55% of the patients showing sac shrinkage. The latter warrants surveillance in these patients^5^ and the need for solutions for inducing sac shrinkage or reducing type 2 ELs are still under discussion.^5^

Limitations of this study is the retrospective nature resulting in a selecting bias, the availability of short-term outcomes only, which makes the analysis of target vessel patency and sac dynamics difficult and lacking enough numbers to perform such analysis in the follow-up. Nevertheless, the first experiences and outcomes are promising.

## Conclusion

The results of these first experiences using the custom-made fenestrated TREO® Abdominal Stent-Graft System in 4 different medical centers in Europe are promising. In this study, there was a high success rate for the cannulation of the target vessels and placement of the bridging stents through the fenestrations (99%). There was a low short-term mortality and morbidity rate in these patients of which 17% had previous AAA repair. Mid-term and long-term follow-up data are needed to evaluate endograft durability and performance.
